# Facilitators and “deal breakers”: a mixed methods study investigating implementation of the Goal setting and action planning (G-AP) framework in community rehabilitation teams

**DOI:** 10.1186/s12913-020-05651-2

**Published:** 2020-08-25

**Authors:** Lesley Scobbie, Edward A. S. Duncan, Marian C. Brady, Katie Thomson, Sally Wyke

**Affiliations:** 1 Nursing, Midwifery and Allied Health Profession Research Unit, Govan Mbeki Building, Glasgow Caledonian University, Cowcaddens Road, Glasgow, G4 OBA, Scotland; 2 Nursing, Midwifery and Allied Health Professions Research Unit, Unit 13 Scion House, University of Stirling Innovation Park, Stirling, FK9 4NF Scotland; 3 Institute of Health and Wellbeing, University of Glasgow, 1 Lilybank Gardens, Glasgow, G12 8R2 Scotland

**Keywords:** Goal setting, Stroke, Community rehabilitation, Implementation, Mixed methods

## Abstract

**Background:**

High quality goal setting in stroke rehabilitation is vital, but challenging to deliver. The G-AP framework (including staff training and a stroke survivor held G-AP record) guides patient centred goal setting with stroke survivors in community rehabilitation teams. We found G-AP was acceptable, feasible to deliver and clinically useful in one team. The aim of this study was to conduct a mixed methods investigation of G-AP implementation in diverse community teams prior to a large-scale evaluation.

**Methods:**

We approached Scottish community rehabilitation teams to take part. Following training, G-AP was delivered to stroke survivors within participating teams for 6 months. We investigated staff experiences of G-AP training and its implementation using focus groups and a training questionnaire. We investigated fidelity of G-AP delivery through case note review. Focus group data were analysed using a Framework approach; identified themes were mapped into Normalisation Process Theory constructs. Questionnaire and case note data were analysed descriptively.

**Results:**

We recruited three teams comprising 55 rehabilitation staff. Almost all staff (93%, 51/55) participated in G-AP training; of those, 80% (*n* = 41/51) completed the training questionnaire. Training was rated as ‘good’ or ‘very good’ by almost all staff (92%, *n* = 37/41). G-AP was broadly implemented as intended in two teams. Implementation facilitators included - G-AP ‘made sense’; repetitive use of G-AP in practice; flexible G-AP delivery and positive staff appraisals of G-AP impact. G-AP failed to gain traction in the third team. Implementation barriers included - delays between G-AP training and implementation; limited leadership engagement; a poor ‘fit’ between G-AP and the team organisational structure and simultaneous delivery of other goal setting methods. Staff recommended (i) development of training to include implementation planning; (ii) ongoing local implementation review and tailoring, and (iii) development of electronic and aphasia friendly G-AP records.

**Conclusions:**

The interaction between G-AP and the practice setting is critical to implementation success or failure. Whilst facilitators support implementation success, barriers can collectively act as implementation “deal breakers”. Local G-AP implementation efforts should be planned, monitored and tailored. These insights can inform implementation of other complex interventions in community rehabilitation settings.

## Background

High quality goal setting in stroke rehabilitation is vital [1, 2], but highly challenging to deliver [3–6]. The G-AP framework was co-produced [7] by researchers and rehabilitation staff to guide goal setting practice with stroke survivors in community rehabilitation settings [8]. Evidence and theory based [9, 10], G-AP informs a person-centred approach to the setting and pursuit of rehabilitation goals in four key stages: (i) goal negotiation & setting, (ii) action planning & coping planning, (iii) action and (iv) appraisal, feedback & decision making. It is designed to be delivered flexibly by multi-disciplinary rehabilitation staff and tailored to local contexts. G-AP training (online and face to face) prepares staff to deliver G-AP in practice. Stroke survivor’s goals, action plans and progress are recorded in the G-AP stroke survivor held record.

We conducted an initial evaluation of G-AP implementation in one community rehabilitation team [11]. Our findings indicated that G-AP was clinically useful, feasible to deliver and broadly acceptable to stroke survivors and staff. Staff made helpful recommendations to improve the G-AP training and stroke survivor held record. However, this small feasibility study included only eight stroke survivors and eight rehabilitation staff. Furthermore, the participating team was involved in G-AP development [8], so was arguably better placed to deliver G-AP than teams with no prior exposure.

Implementation of complex interventions like G-AP in health and social care settings is challenging, even with robust evidence to support their effect [12]. Features of the practice setting (or context) influence the extent to which interventions can be successfully implemented and embedded [13–15]. Interventions successfully implemented in some settings may fail to gain traction in others [16] or be de-implemented over time [17]. Complex interventions interact with practice settings at multiple levels, including the service level (or team in which the intervention is delivered), staff level (team members delivering the intervention) and patient level (those receiving the intervention) [18].

Process evaluations can help us to understand the implementation of complex interventions in different contexts [19], and are particularly useful if they use and test implementation theory [20]. Normalisation process theory (NPT) has been widely used in health services research to investigate and explain the processes and organisational issues that influence implementation of new innovations into practice [21]. NPT includes four explanatory constructs that conceptualise the ‘work’ of implementation: (i) *coherence* (understanding and making sense of the intervention), (ii) *cognitive participation* (commitment to, and engagement with, the intervention), (iii) *collective action* (enacting the practices required of the intervention) and (iv) *reflexive monitoring* (reflecting on the effects of the practice; reconfiguration of how practice is enacted) [22]. Successfully engaging in the ‘work’ to make sense of the innovation, to engage proactively with making it work in one’s own setting, to take action to deliver the innovation and to reflect on its success and take action to improve it, is reported to support the successful implementation and embedding of interventions into practice [21, 23].

### Study aim

We aimed to investigate G-AP training and implementation in community rehabilitation teams with no prior exposure to G-AP, using NPT to inform data analysis. We asked: Does G-AP training prepare staff to implement G-AP in practice? Can different community rehabilitation teams implement G-AP as intended? And, what are the barriers and facilitators to G-AP implementation in practice? Stroke survivors’ experiences of G-AP implementation were also investigated and are reported elsewhere [24].

## Methods

### Study procedure and design

**Fig. 1:**
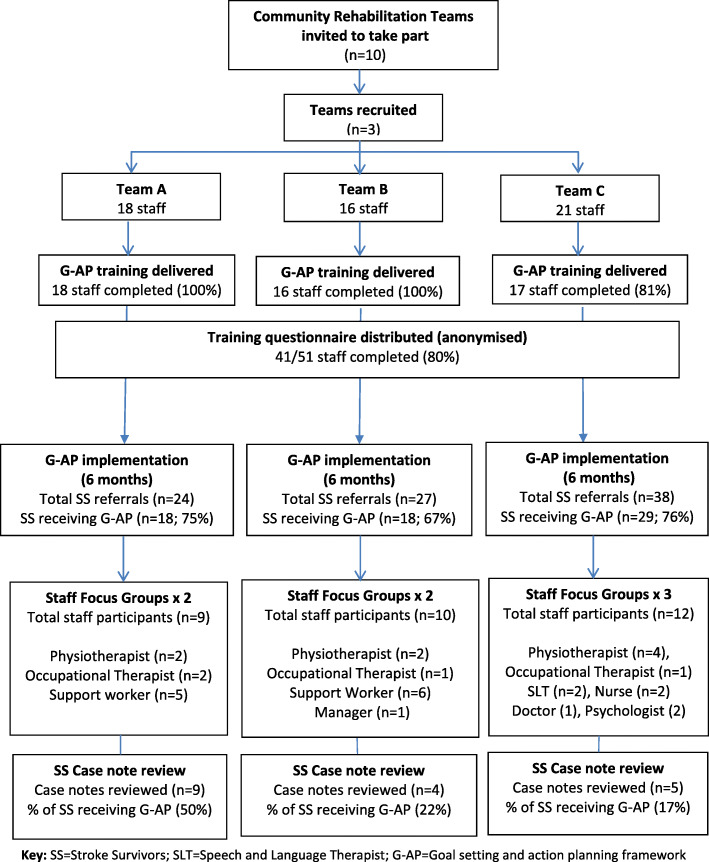
Study procedure and participants. Key: SS=Stroke Survivors; SLT = Speech and Language Therapist

Our evaluation was conducted using mixed methods in Scottish community rehabilitation teams from February to July 2014 (see Fig. 1. Study procedure and participants). The standards for quality improvement reporting excellence (SQUIRE) [25] and the standards for reporting qualitative research (SRQR) [26] were used to inform the conduct and reporting of this study.

### The context: participating teams and usual goal setting practice

Ten teams identified through a previous UK survey [27] and located within a 60 mile radius of the research base, were invited to take part. Three teams agreed; staff shortages and commitment to other projects were the main reasons teams declined.

Team A and B included 18 and 16 multidisciplinary staff respectively, comprising five professional groups – team manager, physiotherapy, occupational therapy, work and training advisor and support workers. The teams were part of a new integrated health and social care stroke specific service. These teams were treated separately for the purposes of this study as they (i) were located in different day centres, (ii) covered different geographical areas, (iii) were managed separately, and (iv) had different staff (although a small minority operated across both teams).

Team C included 21 multidisciplinary staff, comprising seven professional groups – rehabilitation doctors, occupational therapy, physiotherapy, rehabilitation assistants, speech and language therapy, dietician and specialist nurses. This was a long established, consultant led NHS team, providing rehabilitation to a range of people with neurological conditions, including stroke (see Additional File 1: Team details).

Goal setting was reported by staff to be a highly valued and integral aspect of rehabilitation practice within all three teams prior to the start of the study, and was supported by integrated case notes (i.e. documentation from all disciplines were included within the notes) and goal review meetings (see Additional File 2: Usual goal setting practice). Key differences between G-AP informed and ‘usual’ goal setting practice are summarised in Table 1.

**Table 1:** G-AP informed versus usual goal setting practice

G-AP informed goal setting practice	Usual goal setting practice
Theory driven approach incorporating: (i) goal negotiation & setting, (ii) planning (iii) action and (iv) appraisal, feedback & decision making	Various approaches: OT’s used COPM in all teams; SLT’s used Care Aims in Team 3; other disciplines used own approach
Use of the stroke survivor held G-AP to record goals, plans and progress	No stroke survivor held record to record goals, plans and progress
Action plans agreed; coping plans developed to overcome anticipated barriers; confidence to complete plans assessed	Action planning variable; coping plans not routinely discussed; confidence to complete plans not assessed
Ongoing goal and action plan review, appraisal and feedback	Goal review typically at the end of the intervention period

*OT* Occupational Therapist, *COPM* Canadian Occupational Performance Measure [28], *SLT* Speech and Language Therapist; Care Aims: person centred outcomes focused approach (https://careaims.com/about-care-aims/)

### G-AP training

G-AP training consisted of an online training module [29] and a face to face training day delivered to each team by LS and another researcher (SB or ED). *Online training* focused on the theoretical underpinning of G-AP and included case study examples to illustrate its use in practice. *Face to face training* included presentations about each G-AP stage followed by group work based on case study material (see Additional File 3: Outline of G-AP training day). Evidence based techniques including role play, information provision, feedback and modelling [30] were used to enhance staff knowledge, skills and confidence to implement G-AP in practice. Training highlighted the key components of G-AP to be delivered to each stroke survivor (see Table 2); but staff were encouraged to tailor delivery of G-AP to individual stroke survivors as required.

**Table 2:** Key components of G-AP

In partnership with the stroke survivor:	
• Identify stroke survivor’s needs, preferences & priorities	
• Agree a specific goal(s)	
• Agree an action plan(s) for each goal	
• Consider a coping plan if barrier anticipated	
• Measure confidence to complete the action plan	
• Appraise outcome of each action plan & goal progress	
• Give feedback & decide what to do next	

*The G-AP record included carbon copies of documented goals, plans and progress for removal and insertion into stroke survivor’s integrated case notes

### Data collection

To support our implementation study, three data collection methods were used: *1. G-AP training evaluation:* A bespoke online training evaluation was developed using Survey Monkey Inc. to evaluate the online and face to face G-AP training (see Additional File 4: G-AP training evaluation). Anonymised evaluations were completed by staff within a week of completing the G-AP training, *2. Staff focus groups*: We aimed to conduct at least one focus group per team following the implementation period, with representation from all professional groups. The focus group topic guide included questions about usefulness of the G-AP training, experiences of using G-AP in practice, factors that facilitated or hindered implementation and views about its impact (if any) (see Additional File 5: Focus group topic guide) and 3*. Case note review*: Case-note data were extracted in each team base [LS]. Data relevant to implementation of key components of G-AP were extracted from stroke survivor’s case notes (which included carbon copies of the G-AP record) and tabulated (see Additional File 6: Case note data extraction table).

### Data analysis

Each of our three data sets were analysed separately. No formal integration of findings was planned as our data collection methods and analysis primarily focused on individual research questions [31].

Training questionnaire data were analysed using descriptive statistics; open ended responses were collated and summarised [LS].

We analysed focus group data in two stages. In Stage 1 we used a ‘Framework’ approach [32, 33] to explore themes within and between teams. Focus group transcripts were checked against audio recordings and anonymised [LS] to ensure the accuracy of, and established familiarisation with, the whole data set. Anonymised transcripts were imported into QSR International NVivo 10 qualitative data analysis software to facilitate data management. Three transcripts were read to identify broad expected and novel themes informed by the study research questions [LS]. The broad thematic framework was reviewed and agreed by the project team then applied to the remaining transcripts. Following this, data within each broad theme were reviewed and coded into sub themes [LS]. The developing thematic framework was then reviewed and further refined [LS, ED]. Redundant sub-themes were removed, overlapping themes merged and others relabelled to better reflect the data contained within them. We then discussed and approved the final thematic framework (see Additional File 7. Final thematic framework). In Stage 2 we developed an initial definition for each NPT construct, with terms adapted to reflect the nature of our study (see Additional File 8: G-AP NPT coding framework). Two team members [LS, KT] independently mapped sub-themes onto NPT constructs; then together reviewed mapping decisions. Mapping discrepancies were identified and resolved through a process of discussion to consensus (see Additional File 9: Mapping of themes to NPT constructs).

Case-note data were tabulated and analysed descriptively [LS].

### Approvals

Ethical approval was obtained from the West of Scotland Research Ethics Service (ref no: 12/WS/0292) and University of Stirling School of Nursing, Midwifery and Health Research Ethics Committee. Research and Development approval was obtained from participating health boards. Staff provided informed written consent to participate in focus groups. Stroke survivors provided informed written consent for their case notes to be reviewed.

### Researcher characteristics and reflexivity

Development of the G-AP framework was led by LS, an occupational therapist with experience working in community rehabilitation settings. LS was trained in the use of qualitative methods and had successfully used the Framework approach in a previous study [11]. Maintaining a neutral stance was a priority for the research team throughout. LS completed reflexive diaries and field notes following each focus group; these were discussed research team meetings to support a transparent and objective approach to data collection, analysis and interpretation.

## Results

### Study participants (see Fig. 1: study procedure and participants)

#### Stroke survivors

G-AP was implemented with the majority (73%, 65/89) of stroke survivors referred to participating teams. The following reasons were reported for not implementing G-AP: short rehabilitation input (< 3 visits) (*n* = 7); no goals identified (*n* = 3); stroke survivor declined rehabilitation (*n* = 2); onward referral to another service (*n* = 2), stroke survivor unable to participate in rehabilitation (*n* = 4) and reason not reported (*n* = 6). Eighteen stroke survivors consented to their case notes being reviewed. Demographic details of stroke survivors are reported elsewhere [24].

#### Rehabilitation staff

The vast majority (*n* = 51/55; 93%) of staff across the three teams completed the G-AP training and delivered G-AP in practice. In Team C, the rehabilitation consultant (team lead), consultant nurse and two rehabilitation assistants (*n* = 4) were unable to attend due to competing clinical priorities (the nurse attended a short training ‘top up’ session). More than half (31/51, 61%) of the staff across the three teams took part in the focus group discussions. For logistical reasons, seven small focus groups were convened rather than one per team as planned. All health and social care staff groups were represented with the exception of the Team A manager (vacant post) and the Team C dietician (unavailable). Focus groups lasted from 60 to 90 min each and took place in team base.

### G-AP training evaluation

Eighty percent (*n* = 41/51) of staff who participated in training completed the G-AP training questionnaire.

#### Online training responses

Eighty percent (*n* = 33/41) of staff rated the online G-AP training as ‘Good’ or ‘Very Good’; 97% (*n* = 40/41) reported that its content was relevant to practice and 95% (*n* = 39/41) reported they would recommend the training to others. The majority (n = 39/41; 95%) of staff found the case study material helpful. Most staff (*n* = 27/41; 66%) reported the online training took 1–2 h to complete and that computer access to complete it was easy (*n* = 38/41; 93%). Most staff (*n* = 29/41; 71%) found the online training easy to use; but some (*n* = 6/41; 15%) had difficulty navigating through it and printing the completion certificate (*n* = 11/41; 27%). Free text responses highlighted differing perspectives of the online training. One staff member explained, “*Personally, web-based training doesn’t work for me. I would have preferred just learning on the* [face to face training] *day*” (Response 1; Q9). Others had a more positive view commenting that it was, “*Particularly* [useful] *for new staff and students*” (Response 6; Q10) and that, “*It* [online training] *gives a clear overview of the theory and process of G-AP*” (Response 18; Q12).

#### Face to face training responses

Ninety-two percent (*n* = 37/40) of staff rated the face to face G-AP training as ‘Good’ or ‘Very Good’ and said they would recommend the training to others. Almost all (40/41, 98%) staff reported that the training content was relevant to their practice. Most (*n* = 32/40; 80%) said the length of the training was ‘about right’; but some (*n* = 8/40; 20%) felt it was ‘too long’. The vast majority (*n* = 37/40; 92%) of staff found role-play’s helpful; however, some did not and suggested discussion of case vignettes as an alternative. In general, staff valued opportunities for discussion, “*I think it’s always helpful to hear about real life examples and to have the opportunity to discuss and ask questions.*” (Response 9; Q20). Staff reported that they were either ‘somewhat confident’ (25/40, 62%) or ‘very confident’ (15/40, 38%) that the G-AP online and face to face training had prepared them to use G-AP in practice. One staff member commented, *“The training day gave me the confidence to start using it* [G-AP] *straight away*” (Response 2; Q19). The majority (27/40, 68%) of staff were ‘very committed’ to using G-AP in practice. However, a small minority (2/40, 5%) reported they were ‘not at all’ committed to using G-AP in practice and would not recommend the training to others (3/40, 8%).

### Case note review

Eighteen integrated case notes, incorporating duplicate sheets of the G-AP record, were reviewed.

*Team A* (*n* = 9): G-AP was delivered as intended in 5/9 cases; minor inconsistencies were noted in 4/9 cases (for example, measuring confidence to complete action plans was inconsistently recorded). Documented goals and plans were person centred (for example, Goal “*To use belly breathing to manage my anxiety in real situations.”* Action plan: “*Practise belly breathing before I leave to go to the local shop and when I’m in there*.” (Case Note A11).

*Team B* (*n* = 4): G-AP was delivered with minor inconsistencies in 4/4 cases (for example, action plans were documented for some but not all goals). Documented goals and plans were person centred (for example, Goal: “*To spend more time with my brother*.” Action plan: “*Ask my brother if he wants to play a game of pool this week.”* (Case note B13).

*Team C (n = 5)*: G-AP was delivered with significant inconsistencies in 5/5 cases. There was no evidence of G-AP being delivered by one or more staff in 3/5 cases. Goals and action plans were typically recorded in profession specific sections of the case notes, rather than in the G-AP record. Those goals that were documented were person centred (for example, Goal: “*To walk to supermarket and get the messages* [shopping] *on my own.*” Action Plan: “*Practice walking to the end of the street and back.*” (Case Note C1). Goals addressing stroke survivor psychological or emotional issues were not documented in any case notes. Psychology notes were not available for review within the integrated note as they were filed separately.

### Implementation of the G-AP framework

**Fig. 2:**
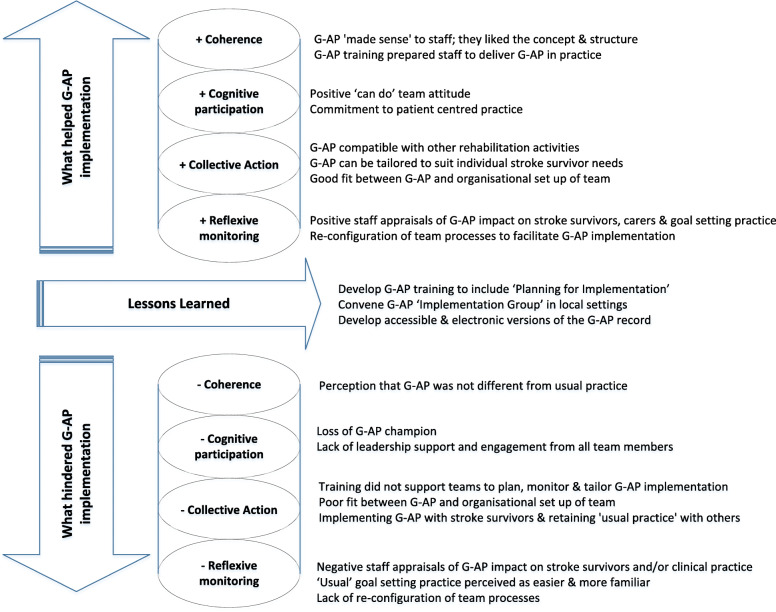
Main themes and sub themes linked to Normalisation Process Theory constructs

Three overarching themes captured staff perspectives of G-AP implementation (i) what helped G-AP implementation, (ii) what hindered G-AP implementation and (iii) lessons learned. In the following sections, each theme and related sub themes are reported under Normalisation Process theory constructs - coherence, cognitive participation, collective action and reflexive monitoring. Supporting data are presented in a table at the end of each section. Figure 2 presents an overview of the relationship between themes, subthemes and Normalisation Process Theory constructs.

### Coherence (making sense of G-AP)

Staff in all three teams said that G-AP made sense to them; they liked the concept and structure of it (Quote 1&2). G-AP training was positively received. Staff reported that it helped them to understand G-AP and how to deliver it in practice (Quote 3). However, it was felt that the best way to gain confidence with G-AP was to actually use it (Quote 4). Most staff viewed G-AP as distinct from their usual goal setting practice (Quote 5). However, a marginal view was that there was little difference (if any) between the two (Quote 6) Table 3.

**Table 3:** Coherence: supporting data

Quotes 1–6	
1*: “I think the whole idea* [of G-AP] *is absolutely brilliant!” (*Occupational Therapist; Team B)	
2: *“I really like the structure, the staged cycle of G-AP.”* (Physiotherapist; Team B)	
3: “*There were loads of really good patient scenarios* [in the training] *and I really understood the* [G-AP] *system and how to use it.”* (Occupational Therapist; Team C)	
4: “*There’s only so much of training you can get, it’s best just to get on with it I think.”* (Support worker; Team A)	
5: *“I suppose that what’s always been drilled into* [us]*; setting your SMART goals, it’s got to be something really specific and measurable. Whereas I suppose with the G-AP it is a bit more patient focussed and it’s like, what exactly do they want to do and how do we put it in their kind of words.”* (Physiotherapist, Team 3)	
6: *“G-AP is something we already do in a different name.” (Questionnaire Response 1; Q19).*	

### Cognitive participation (commitment to and engagement with G-AP)

Staff viewed G-AP as relevant and integral to their clinical practice (Quote 7). This, combined a positive team approach (Quote 8), valuing patient centred practice and having existing goal setting skills to build on supported cognitive participation. However, commitment to and engagement with G-AP was compromised with frequent staff turnover and loss of a G-AP ‘champion’ from the team (Quote 9). Commitment to G-AP was further compromised if usual goal setting practice was perceived as better (Quote 10) or more familiar (Quote 11). It was acknowledged that lack of commitment from *all* team members could compromise successful implementation (Quote 12) Table 4.

**Table 4:** Cognitive Participation: supporting data

Quotes 7–12	
**7.** *“G-AP felt like something that was a key process, that you could use from the start right through.”* (Nurse; Team C)	
8. “*We work here as a team as to what’s best for the service user* [stroke survivor], *you know, when you start from that point, then everything’s achievable.”* (Support worker; Team B)	
9. *“M* [nurse; Team C] *is retiring next month. I can see this being a problem … she has been the main driver behind G-AP.”* (LS field note entry; 07/03/2014)	
10. *“I’d probably go with the Canadian* [Occupational Performance measure] *I think* [rather than G-AP] *because it’s very quick to fill out, it’s relatively simple and it gives you that outcome measure which is obviously something that we can use to back up why we’re doing what we’re doing.”* (Occupational Therapist; Team C)	
11. *“Until you’re familiar with doing it* [G-AP], *it feels awkward, you know, so you tend to stick with what’s comfortable, what you’re used to* [usual practice]. *It’s like putting on your old jeans compared to a nice new pair of jeans that feel a bit awkward.”* (Psychologist; Team C)	
12. *“It would be convincing every single team member that you need to be enthusiastic and motivated, that they’re going to get what they need out of it* [G-AP].” (Nurse; Team C)	

### Collective action (the ‘work’ staff do - individually and collectively - to deliver G-AP)

Successful collective action was critical to ongoing G-AP implementation. The compatibility of G-AP with other rehabilitation activities and its flexible format supported staff to work, both individually and collectively, to deliver G-AP in their own setting (Quote 13 & 14). Tailoring G-AP to accommodate individual stroke survivor needs supported staff to deliver G-AP with the majority of stroke survivors referred to the team (Quote 15).

The organisational set up of teams played a critical role in facilitating or inhibiting collective action. Scheduling processes that maintained continuity between staff and stroke survivors helped staff to build confidence delivering G-AP with the same person over time (Quote 16). In stroke specific teams, consistent and frequent delivery of G-AP with stroke survivors on a day to day basis helped staff to embed G-AP in their routine practice (Quote 17). However, delivering G-AP to stroke survivors whilst retaining usual goal setting practice with other patient groups in the mixed neurological team prevented staff from gathering momentum using G-AP (Quote 18). Staged team assessment and internal referral processes could result in delays (of up to six weeks) between G-AP training and implementation. This de-energised implementation efforts at the outset. Additionally, staff waiting lists resulted in team members initiating rehabilitation input at different times, thus fostering a unidisciplinary rather than interdisciplinary approach to G-AP implementation (Quote 19 &20). Mandatory service requirements to use other goal setting tools alongside G-AP also resulted in unhelpful duplication (Quote 21). Collectively, these organisational barriers could at best frustrate, and at worst impede collective action. Staff acknowledged that overcoming barriers would require fundamental organisational change and leadership support (Quote 22 & 23) Table 5.

**Table 5:** Collective Action: supporting data

Quotes 13–23	
13. *“But it was quite helpful to do that* [use Talking Mats at the goal negotiation/ goal setting stage]*, yeah. I think that* [Talking Mats] *fits in quite well with this* [the G-AP framework]*.”*	
14 *“Some of the other* [goal setting] *systems where it’s too rigid; things must be done by this point, all of you must do this together, the patient must be involved in that part or not and things, there was more flexibility in G-AP than that.”* (Physiotherapist; Team C)	
15. *“In principle, I think there’s always a way you could use G-AP with pretty much anybody,* [be] *cause it’s a framework as opposed to defining somebody.”* (Support worker; Team A)	
16. *“If support workers have continuity with the same client, it gives them experience and confidence* [delivering G-AP in practice].” (Occupational Therapist; Team B)	
17. “*The more you use it* [G-AP] *... it just kind of became a bit more second nature as you saw the whole stages, you kind of saw it unfolding, so I think that helped just using it more, embracing it.”* (Physiotherapist; Team A)	
18. *“I think it’s been quite sporadic* [G-AP implementation] *because we’re just using it for stroke patients and not any other neurological groups. So, there was a bit of kind of two systems going at the same time. If we’re doing the same thing with every single patient, then it probably would have been easier for that to have been routine.”* (Physiotherapist; Team 3)	
19*. “There was certainly a bit of a gap between the training and the first person I saw. Whereas if you’d been bang, straight in there, you know, it might have been more likely to sort of gather momentum.”* (Psychologist; Team C)	
20. “*It’s done* [G-AP implementation] *in isolation and individual disciplines. It’s not done as a team, because people are seen at different times.”* (Doctor; Team C)	
21. “[To use Malcomess Care Aims] *you’re going to have to duplicate over your goals from what you’ve set during the G-AP. We would re-write them, so that’s duplication.*” (Speech and Language Therapist; Team C)	
22*. “I suppose the whole structure and organisation of the team* [would have to change to support G-AP implementation]*. And agreement within, you know, how that works within our service.”* (Nurse, Team C)	
23. *“If he’s not behind it* [the rehabilitation consultant] *then people start to lose motivation. He definitely needs to be key to the process*.” (Physiotherapist, Team C)	

### Reflexive monitoring (assessing the effects of G-AP; reconfiguration of how G-AP is enacted)

Staff reported mostly positive, but some negative appraisals of G-AP impact which influenced ongoing implementation efforts. Staff across all teams reported positive impacts of G-AP on stroke survivors and goal setting practice. A strong theme reported was that G-AP supported stroke survivors take ownership and control of the process (Quote 24) and to identify personal goals and action plans, which in turn enhanced their focus and motivation (Quote 25). Use of the G-AP record helped stroke survivors to gauge their progress and feel encouraged about progress made (Quote 26). Staff reflections suggested that G-AP supported an enhanced interdisciplinary, patient centered and goal focused approach to practice (Quote 27). Additionally, the structure of G-AP reminded staff to implementation of all stages of the process (Quote 28). These perceived positive impacts encouraged ongoing G-AP implementation and mobilised efforts to reconfigure team processes to facilitate use of G-AP in day to day practice. This included introducing review of the G-AP record in goal meetings and creating mentoring opportunities for staff less confident using G-AP in practice (Quote 29).

Although marginally held views, two potential negative impacts of G-AP were reported, (i) introducing G-AP to stroke survivors experiencing complex emotional issues required careful handling and could be counterproductive (Quote 30) and (ii) G-AP could result in prioritisation of ‘behavioral’ goals over those that focused on ‘thoughts’ or ‘emotions’ (Quote 31). These concerns discouraged implementation efforts and reinforced a view that usual practice was satisfactory or better.

Reflexive monitoring informed ‘lessons learned’ within teams. There was consensus that implementation required local planning and decision making about *who* should implement *what* aspect of G-AP and *when* based on the skill mix and availability of team members. Staff suggested this could usefully be incorporated into G-AP training (Quote 32) and that setting up a group to support and monitor local G-AP implementation would be helpful (Quote 33). Finally, staff recommended that different formats of the G-AP record should be available to suit individual stroke survivor needs and preferences. This included having an accessible version for stroke survivors with aphasia and an electronic version to minimise the need for duplicate notes (Quote 34) Table 6.

**Table 6:** Reflexive monitoring: supporting data

Quotes 24–34	
24. “*I think there was ownership for her* [the stroke survivor], *this is my goal, here’s what I want to set out and do, you guys* [rehabilitation staff] *help me achieve it*.” (Support Worker; Team A)	
25. “*I can think of one particular woman who wanted to work on a computer* [to do online shopping], *and we sat down, we talked through what was the best way to break down this goal so that she was able to get the focus and see what she needed to do to work this computer. And it was amazing. Over the period of a few weeks, she went from not really knowing what to do, to being competent totall*y!” (Support Worker; Team B)	
26. “*It’s a really good tool* [the G-AP record] *to record the journey that somebody is going through, especially if they’ve got any sort of memory problems. You’ve got proper evidence of where they came from. You can say, ‘Oh a couple of weeks ago you couldn’t do that, but now look!’ So, yes, it definitely added value*.” (Support Worker; Team A)	
27. “*Well before* [G-AP] *the goals would be fairly uni-professional or they would be highlighted as certain professional goals, so that would be a sort of physio* [therapy] *type of goal, or an OT* [Occupational Therapy] *type of goal*. [Now] *we’d think a bit more about the patient’s goal and how they’re going to achieve that and how we would fit into that, rather than how they would do their physio goal, if that makes sense.*” (Physiotherapist; Team B)	
28. “*As someone who’s done goal setting for years, it’s quite helpful to think through, ‘Right, negotiating the goals, setting them, then how are we going to achieve them, what are the barriers* etc.*’, I think the structure has been quite helpful. It’s very easy to skip steps, and you’ve not talked about what the barriers could be or you forget that ‘Right, we’ll come back and review this’*.” (Physiotherapist; Team B)	
29. “*When I was actually doing it* [G-AP] *with a client, I asked one of the support workers if they wanted to come and sit with me because I knew that that worker would probably spend more time with that person than me or anybody else. And that seemed to work quite well. I knew that support worker then taught another support worker. So I don’t think it has to be a therapist, it could be a champion or - I don’t know what the right word is, a mentor*.” (Occupational Therapist; Team B)	
30. “*I mean, if I’d had a stroke, and I’m in a terrible mess, and I’m upset and somebody sits down ‘Right now what are your goals?’ you know. It can be very patronising to make a goal, you know, as well and it can diminish, their experience... if they’re in the middle of telling you about these experiences that are hugely traumatic, you can’t suddenly say ‘Right, what are you going to do for next week then just before you go?’ It’s like I’ve not listened to them if I go in and do that*.” (Psychologist 1; Team C)	
31. “*When it was more working at a sort of emotional level, or at level of thoughts, it was difficult to set meaningful goals in relation to that*.” (Psychologist 2, Team C)	
32. “*Perhaps if there had been some encouragement of ‘Right, you’ve got the process now, you know the principles, this is the* [G-AP] *folder, now sit down as a team and then think about it’ - that was maybe the one bit that was missing [in the G-AP training*].” (Physiotherapist; Team B)	
33. “*It’d be worthwhile almost to have some sort of like supervision type group that you could go along to; otherwise you’re sort of using it* [G-AP] *in isolation*. *You’re not really getting a chance to speak to people about how it’s going and what’s trouble, what’s difficult, what’s going right, what’s going wrong*.” (Psychologist; Team C)	
34. “*This* [the G-AP record] *could be something that worked really well electronically; but in paper you’re just duplicating more work and it just kind of made it more time. This could be an App; it would be a perfect App*.” (Support worker; Team A)	

## Discussion

Our mixed methods study provides novel insights into the implementation of G-AP across diverse community rehabilitation teams. G-AP online and face to face training prepared staff to deliver G-AP in practice; but did not support them to plan, monitor and tailor G-AP implementation within their local teams. G-AP was broadly implemented as intended in two teams, but failed to gain traction in the third. Barriers to implementation were multi-factorial and collectively could act as implementation “deal breakers”. Staff recommended that the G-AP record should be available in electronic formats and accessible to stroke survivors with aphasia.

### Barriers and facilitators to G-AP implementation in clinical practice

Varying degrees of implementation success and failure is a common theme in studies evaluating the implementation of complex interventions in health settings [34–37]. G-AP was no exception to this. Consistent with Normalisation Process Theory [21–23, 38], we found that the ‘work’ of G-AP implementation - coherence, cognitive participation, collective action and reflexive monitoring - was ongoing, interconnected and affected by the presence of barriers and facilitators within each team.

Team A and Team B benefited from most identified facilitators and few barriers. On the whole, staff in both teams were able to work (individually and collectively) to implement G-AP as intended. This resulted in positive appraisals of G-AP impact, which in turn encouraged reconfiguration of team structures and process to support ongoing local implementation. Team C reported fewer facilitators and considerably more barriers, many of which were related to the organisational set up of the team. Collectively, these barriers disrupted collective action and acted as implementation “deal breakers” by impeding or obstructing staff efforts to implement G-AP. This resulted in more neutral or negative appraisals of G-AP impact, which in turn discouraged reconfiguration of team structures and processes to overcome implementation barriers.

### Understanding implementation failure

#### Intervention and service setting ‘fit’

The unfolding interaction between an intervention and the setting in which it is delivered is integral to the implementation process [39–41]. Services inevitably need to adapt, to varying degrees, to create a workable fit between the two [39, 42]. Interventions requiring major organisational or system change are more likely to result in non-adoption or abandonment [17]. G-AP had a workable fit with Team A and B’s established organisational structures and clinical processes. Although some adaptations were required, it did not necessitate fundamental organisational change. However, the fit between G-AP and Team C’s established organisational structures and clinical processes was problematic. Staff recognised that successful implementation would require fundamental changes to the team’s organisation (for example, to reduce delays between G-AP training and implementation) and clinical processes (for example, to remove the mandatory requirement to use other goal setting tools). It would also require G-AP to be delivered to all patient groups, rather than restricted to stroke survivors. These changes were not forthcoming during the implementation period.

#### Effective agents of change

Commitment and engagement from those in a position to effect change is essential to successful implementation [40–42]. So too is the role of ‘champions’ who can motivate and tailor implementation efforts in local settings [40, 43]. Our findings suggested that in Team C, competing priorities experienced by those in leadership positions and staff changes (resulting in loss of a G-AP champion) may explain to some extent why the necessary changes to organisational structures and clinical processes required to support successful G-AP implementation were not forthcoming. On reflection, we did not make it explicitly clear what our expectations were of those in leadership positions, firstly because we had not fully considered their role during the implementation process, and secondly because we wanted teams to be autonomous as they navigated the implementation process. This may have resulted in team leader’s feeling uncertain about their role in the context of the study, especially when implementation was proving problematic. These are important insights that will inform how we pro-actively engage with team leaders and champions in our future research.

#### Professional identity and scope of practice

The work required to deliver a new intervention should fit with the professional identity and scope of practice of all individual staff [17]. This poses a challenge for G-AP implementation given it is designed to be delivered by multi-disciplinary teams comprising different professional groups [11, 27]. Our findings suggest that G-AP fitted with the professional identity and scope of practice of most, but not all, of the multidisciplinary team. Psychology staff reported that G-AP did not support their practice when working with stroke survivors experiencing complex emotional needs or to set goals targeting emotions or thoughts. This was concerning given the high prevalence of psychological problems after stroke [44] and the critical role of psychologists within stroke rehabilitation teams [45].

There is evidence to suggest that stroke survivors who have complex emotional needs may not benefit from G-AP or other goal setting approaches [5, 11]. However, this has to be balanced against the potential benefits of supporting stroke survivors’ to focus on valued goals and to experience success through action plan completion [11, 46, 47]. G-AP, like all rehabilitation interventions, should be delivered using a flexible, person centred approach taking a range of factors (including stroke survivor’s emotional state) into account. Future developments to G-AP training will highlight these important points. Further consideration will also be given to whether G-AP is likely to challenge individual team member’s professional identity and scope of practice, with a view to addressing concerns prior to implementation in local settings.

### Supporting implementation success

#### Planning for implementation

A recent systematic review by Bird et al. (2019) [48] concluded that education interventions on their own were not effective in translating evidence into stroke rehabilitation settings; but multi-component interventions including education, implementation facilitation (e.g. access to a mentor; site specific performance feedback) and local tailoring (e.g. consideration of local barriers and facilitators) were effective. In their review of systematic reviews investigating implementation of e-health interventions, Ross et al. (2016) [41] described planning for implementation as a ‘critical step’. Consistent with these findings, staff in all teams recommended that G-AP training should support staff to develop a local ‘G-AP implementation plan’ and to convene a ‘G-AP implementation group’ to monitor support on-going local implementation. Further developments to G-AP training combined with development of implementation support strategies will be informed by these important findings.

#### Web-based resources to support training and implementation

The international stroke recovery and rehabilitation round table collaborative [49] highlighted the importance of web-based resources to support implementation of stroke evidence into practice. An example of this is the successful uptake of the Graded Repetitive Arm Supplementary Intervention (GRASP) [50, 51] which has been supported by a freely available web-based education resource incorporating training materials, supporting resources and patient demonstration videos [52–54]. Staff recommended that the G-AP record should be available in electronic formats and accessible to stroke survivors with aphasia. We envisage co-producing these additional resources with stroke survivors and staff and having them freely available within a G-AP web-based resource that would include - G-AP publications, online training, practice manuals with supporting video material and implementation support tools.

### Implications for research and practice

Our findings, combined with evidence-based recommendations made in the aforementioned studies [41, 48, 49], will inform future enhancements to G-AP training and implementation strategies (see Table 7). This will be a focus of our ongoing research, the overall aim of which is to fully develop G-AP as a complex intervention (including training, supporting resources, implementation tools and strategies) ready for implementation and evaluation in a suitably designed large scale study.

**Table 7:** Recommendations to enhance to G-AP training and implementation

Evidence-based recommendations	Relevance to enhancing G-AP training and implementation
**1. Support teams to consider the effects of the intervention on existing systems and work practices**	Develop implementation tools to support teams to pro-actively consider how G-AP implementation will effect and interface with: • Existing team structures and clinical practices (e.g. documentation processes, goal review meetings, appointment scheduling etc.).
**2. Identify and engage with implementation stakeholders and ‘champions’**	Throughout the team recruitment, training and implementation process: • Secure ‘buy in’ of key stake holders (e.g. team leaders; managers). • Identify and support local G-AP champion(s).
**3. Plan implementation, including delineation of roles and responsibilities; consider team members’ professional identity and scope of practice**	Develop G-AP training to support teams to consider *who* will implement *what* stage of G-AP and *when.* For example: • Which team member(s) will introduce G-AP? • When will this take place; before/ after initial assessments? • Which team member(s) will negotiate and set goals? • Who will support stroke survivors with aphasia? • What role will rehabilitation assistant’s play? • How will team members work together to implement G-AP?
**4. Provide ongoing education and training to all those involved**	• Develop G-AP web-based resource, providing easy access to G-AP (i) supporting evidence (ii) online training (including webinars) (iii) practice manual and supporting video material; (iv) paper based, electronic and aphasia friendly versions of G-AP record and (v) implementation support tools. • Support teams to create mentorship opportunities.
**5. Build in ongoing local monitoring, evaluation and tailoring of implementation**	• Support convening of a local implementation group to monitor and tailor G-AP implementation over time. • Provide site specific performance feedback and access to a mentor during the implementation period. • Encourage local adaptability.

Finally, we believe that the identification of implementation “deal breakers” is an important area of future study. Predicting which teams are likely to implement interventions successfully, and those that are not, could helpfully inform site recruitment to research trials thus reducing the risk of implementation failure. Greenhalgh et al. (2017) [17] have gone some way to addressing this issue in the context of implementation of health and care technologies. Their non-adoption, abandonment, scale-up, spread and sustainability (NASSS) Framework conceptualises implementation (over seven domains) as being (i) simple (straightforward and predictable); (ii) complicated (multiple interacting components/ issues) or (iii) complex (dynamic; unpredictable). Greenhalgh et al. (2017) propose that implementation programmes characterised by complexity across multiple domains are unlikely to become successfully embedded in routine practice. Building on this framework, or development of a similar framework for use in rehabilitation research, is a promising area of future research.

### Strengths and limitations

The importance of systematic development and evaluation of complex interventions for use in stroke rehabilitation settings, including the training components, has been emphasised [55, 56]. This study builds on our established programme of work to develop and evaluate the G-AP framework [8, 9, 11, 27]. Our explicit focus on the development and evaluation of two training formats is a novel and important contribution. We also demonstrated that G-AP could be successfully implemented, with the majority of stroke survivors, in two different community rehabilitation teams. This creates impetus to move on to the next stage of evaluation. Using Normalisation Process Theory enabled us to gain important new insights on G-AP implementation which will inform the development of the G-AP training and implementation strategies, thus optimising the chances of future implementation success. Distinguishing between implementation failure and intervention failure is an important aspect of clinical practice and clinical trials [57]. We believe that this study is essential preparatory work to increase the adoption of G-AP in clinical settings and reduce the chances of implementation failure in our planned evaluation.

There were some study limitations that should be considered. Firstly, we evaluated G-AP implementation over a 6-month period. Studies evaluating the implementation and embedding of complex interventions over a longer term are lacking [38]. Secondly, we reviewed stroke survivors’ case notes to assess if G-AP was implemented as intended. To increase the accuracy of our case note review, we designed a G-AP folder with removable duplicate sheets for filing within the integrated case notes. While the findings of the case note review and focus group data analysis were consistent; we acknowledge that interpretation of case note data can be variable [58] and that inclusion of observational methods or audio recordings of rehabilitation sessions would have been a useful adjunct to our case note review. Finally, the teams included in this study provided open ended duration of rehabilitation input to patients. Many teams delivering stroke rehabilitation in the community will be restricted to providing input for shorter periods [27]. Implementation efforts and challenges may differ in these types of teams.

## Conclusion

Our rigorous mixed methods process evaluation highlights the importance of the interaction between G-AP and the context in which it is delivered. The significance of this interaction is critical to implementation success or failure. Implementation is most likely to be successful in those teams who perceive G-AP as beneficial and who do not require fundamental changes to organisational structures and clinical processes to deliver it. G-AP training should support staff to plan, monitor and tailor local G-AP implementation and the G-AP record should be available in electronic formats and accessible to stroke survivors with aphasia. These findings will inform development of a new G-AP web-based resource prior to a large scale evaluation of G-AP.

## Supplementary information

**Additional file 1.**

**Additional file 2.**

**Additional file 3.**

**Additional file 4.**

**Additional file 5.**

**Additional file 6.**

**Additional file 7.**

**Additional file 8.**

**Additional file 9.**

## Data Availability

The data generated and analysed during this study are not publicly available due to concerns about confidentiality regarding a small sample size and the sensitive nature of the interviews. Instead, quotations are included within tables. We will be happy to discuss the findings or the analysis if any questions should arise.

## Abbreviations

G-AP: Goal setting and Action Planning framework
NPT: Normalisation Process theory
SS: Stroke Survivor
SLT: Speech and Language Therapist
COPM: Canadian Occupational Performance Measure
OT: Occupational Therapist
