# Supplementary material for: Facilitators and “deal breakers”: a mixed methods study investigating implementation of the Goal setting and action planning (G-AP) framework in community rehabilitation teams
Source: BMC Health Serv Res. 2020 Aug 25;20:791. doi: 10.1186/s12913-020-05651-2 (PMC7447562; doi:10.1186/s12913-020-05651-2)
Supplement: Supplementary file 1 — Additional file 1. [file 12913_2020_5651_MOESM1_ESM.docx]

Supplementary File 1: Team details

|  | **Team A** | **Team B** | **Team C** |
| --- | --- | --- | --- |
| **Team type** | Integrated health and social care team | Integrated health and social care team | Neurological rehabilitation team |
| **Team lead/ manager** | Day centre manager &  Therapy lead | Day centre manager &  Therapy lead | Consultant in Rehabilitation medicine |
| **Team base** | Day centre (a) | Day centre (b) | Hospital out-patient department |
| **Patients seen** | Stroke survivors only | Stroke survivors only | People with neurological conditions (including stroke) |
| **Patient age range** | 18+ years | 18+ years | usually < 65 years |
| **Staff numbers *** | 18 | 16 | 21 |
| **Professional groups represented in team** | Occupational therapy  Physiotherapy  Social care assistant  Work & training advisor  (Onward referral to SLT & psychology if required) | Occupational therapy  Physiotherapy  Social care assistant  Work & training advisor  (Onward referral to SLT & psychology if required | Occupational therapy  Physiotherapy  Rehabilitation assistant  SLT  Dietician  Specialist nurse  Doctor  Psychology |
| **Organisation of rehabilitation input** | Day programme  (9.30am-3pm/ 2 days per week) | Day programme  (9.30am-3pm/ 2 days per week) | Sessional  (Typically one hour sessions) |
| **Typical duration of input** | 13-21 weeks | 13-21 weeks | 22 weeks+ |
| **Usual location of rehabilitation sessions** | Day centre  (home/ community venue if required) | Day centre  (home/ community venue if required) | Out-patient unit  (home/ community venue if required) |
| **Typical number of sessions per week** | Multiple sessions per rehabilitation day  (up to 5) | Multiple sessions per rehabilitation day  (up to 5) | Multiple sessions per week  (up to 5) |
| ^*^ Approximate numbers due to staff changes within study period; SLT: Speech and Language Therapy | | | |
