# Supplementary material for: Facilitators and “deal breakers”: a mixed methods study investigating implementation of the Goal setting and action planning (G-AP) framework in community rehabilitation teams
Source: BMC Health Serv Res. 2020 Aug 25;20:791. doi: 10.1186/s12913-020-05651-2 (PMC7447562; doi:10.1186/s12913-020-05651-2)
Supplement: Supplementary file 2 — Additional file 2. [file 12913_2020_5651_MOESM2_ESM.docx]

Supplementary file 2: Usual goal setting practice

|  | **Team A** | **Team B** | **Team C** |
| --- | --- | --- | --- |
| **Is goal setting used?** | Yes - with all patients | Yes - with all patients | Yes - with most patients |
| **Patient involvement in setting goals** | Individual team members set goals with patients | Individual team members set goals with patients | Individual team members set goals with patients |
| **Goal setting methods used** | OT: COPM  Other disciplines: own method | OT: COPM  Other disciplines: own method | OT: COPM  SLT: Care Aims  Other disciplines: own method |
| **Goal setting meetings** | weekly | weekly | weekly |
| **Priority given to goal setting within the team** | high | high | high |
| **Priority given to involving patients in the goal setting process** | high | high | high |
| **Are goal setting activities documented?** | Yes - Social care staff use electronic recording system; NHS staff use paper based recording system | Yes - Social care staff use electronic recording system; NHS staff use paper based recording system | Yes - Integrated rehabilitation record with goal setting section (paper based) |
| **Are patients given an accessible copy of their goals?** | sometimes | sometimes | sometimes |
| **Key:** OT – Occupational therapy; COPM - Canadian Occupational Performance Measure; SLT – Speech and Language therapy | | | |
