# Supplementary material for: Facilitators and “deal breakers”: a mixed methods study investigating implementation of the Goal setting and action planning (G-AP) framework in community rehabilitation teams
Source: BMC Health Serv Res. 2020 Aug 25;20:791. doi: 10.1186/s12913-020-05651-2 (PMC7447562; doi:10.1186/s12913-020-05651-2)
Supplement: Supplementary file 3 — Additional file 3. [file 12913_2020_5651_MOESM3_ESM.docx]

| **Day 1**  **Additional File 3. Outline of G-AP training day** | **Content** | **Behaviour change technique** | **Targeted theoretical behaviour change domains and expected output** |
| --- | --- | --- | --- |
| 1. Introductions & aims of the training day | Introductions, short presentation & questions |  |  |
| 2. The G-AP Framework: An overview and use of the G-AP patient held record including the rationale for its development | Presentation & questions  Present an example of working through the G-AP process *(“I want to eat more healthily”)*  ***Task 1: Individual & Group*** *-* Work through the G-AP process using a personal goal as an example. One or 2 people share their goal examples (Work sheet 1) | **Provide information** about the G-AP framework (overview) and the patient held record with case study examples (**demonstrate** correct use of the framework and patient held record using case study example)  Trainer will **demonstrate** use of the framework using a personal example  Use an easy **graded task** to practice using the framework. Create opportunities for **encouragement/ feedback** and **social support and comparison** as HPs share their goal examples | Staff will have a basic understanding of each stage the G-AP framework and the different ways that the process can unfold in practice **(**k**nowledge, beliefs about consequences, skills).**  Staff will understand the purpose of the patient held record and how it should be completed **(**k**nowledge, beliefs about consequences, skills).**  Staff will successfully work through each stage of the G-AP framework using a personally relevant goal (**skills**, **confidence, motivation, professional role identity, social influences).** |
| **Break** | | | |
| 3. Coming up with the goal: Goal Negotiation and Goal Setting (Stage 1) | Short presentation & questions | **Provide** **information** and **instruction** about implementation of the Goal negotiation and goal setting stage, properties of a good goal and useful tools and strategies to facilitate the process | Staff will understand how to implement Stage 1 of the G-AP framework (**knowledge, skills, professional role identity).** |
| 3b. Coming up with the goal: Goal Negotiation and Goal Setting | ***Task 2: Role Play*** - Work in groups of 3 (Pt/HP/observer). Practice refining the general goal in to a specific goal using 3 or 4 patient scenarios e.g. **(i)**the ‘ideal’ patient **(ii)** the patient that hangs on to “I just want to be back to normal”; **(iii)** the patient with very high expectations; **(iv)** the patient who is emotionally overwhelmed; **(v)** the patent with communication difficulties (Task sheet 2) | **Rehearsal of relevant skills** using **role plays** which become more challenging **(graded tasks).** Create opportunities for **encouragement/ feedback** | Staff will demonstrate competence in setting effective goals **(knowledge, skills, confidence & motivation).**  Staff will be able to implement stage 1 of the framework clinical scenarios of varying complexity (**knowledge**, **skills**, **confidence, motivation** and **professional role identity).** |
| **Lunch** | | | |
| 4a. Planning the steps to achieve the goal: Action Planning, Coping Planning and measuring confidence (Stage 2)  4b. Planning the steps to achieve the goal: Action Planning, Coping Planning and measuring confidence | Short presentation & questions  ***Task 3: Role Play*** *-* Work in groups of 3 (Pt/HP/observer). Planning the steps to meet the goal with **(i)** a patient who wants to take the lead and is spot on; **(ii)** a patient who wants to take the lead and is off the mark; **(iii)** a patient who wants you to take the lead (Task sheet 3) | **Rehearsal of relevant skills** using **role plays** which become more challenging (**graded tasks**). Create opportunities for **encouragement/ feedback** (Abraham & Michie 2008, Michie et al 2008) | Staff will be able to implement stage 2 of the framework in clinical scenarios of varying complexity **(knowledge, skills, confidence,** **motivation** and **professional role identity).** |
| **Break** | | | |
| 5a.Monitoring progress and making decisions: Appraisal, Feedback and Decision making (Stage 3).  5b.Monitoring progress and making decisions: Appraisal, Feedback and Decision making | Short presentation & questions  ***Task 4: Group discussion*** - Use vignettes as a basis for discussion: (i) the ideal patent who’s working the through the process well; (ii) The patient who’s not adhering to the plans; (iii) the patient who’s repeatedly failing in attempts to complete plans (Task sheet 4) | Provide **information** and **instruction** about appraisal, feedback and decision making with a particular emphasis on how to **cope** with and manage goal non-attainment.  **Rehearsal of relevant skills** using **role plays** which become more challenging (**graded tasks**). Create opportunities for **encouragement/ feedback** (Abraham & Michie 2008, Michie et al 2008 | Staff will be able to implement stage 3 of the framework clinical scenarios of varying complexity (**knowledge**, **skills**, **confidence, motivation** and **professional role identity** |
| 6. Using G-AP in your team | Review G-AP key components |  | Staff will know what they have to do to implement G-AP as intended |
