# Supplementary material for: Facilitators and “deal breakers”: a mixed methods study investigating implementation of the Goal setting and action planning (G-AP) framework in community rehabilitation teams
Source: BMC Health Serv Res. 2020 Aug 25;20:791. doi: 10.1186/s12913-020-05651-2 (PMC7447562; doi:10.1186/s12913-020-05651-2)
Supplement: Supplementary file 4 — Additional file 4. [file 12913_2020_5651_MOESM4_ESM.pdf]

G-AP Taining Evaluation

Introduction

Dear Colleague,

The purpose of this survey is to evaluate the G-AP training you have recently participated in.

I would very much like to capture your views about the G-AP training. Please take 5 to 10 minutes to complete this G-AP training survey. The first set of questions refer to the web based G-AP training and the second set to the G-AP training day.

Please be honest when answering the questions - your answers are very important and will help me to improve G-AP training for future participants.

Thank you for taking the time to complete this survey!

Lesley.

The following questions refer to the web based G-AP training ...

Web based G-AP training

***1. Did you complete the web based G-AP training?**

- ○ Yes
- ○ No
- ○ To some extent

Web based G-AP training: Practical Use

***2. Was it difficult to access a computer at work to complete the training?**

- ○ Yes
- ○ No

If 'Yes' can you explain why ...

***3. Did you find the website easy to navigate through?**

- ◯ Yes
- ◯ No

If 'No' can you explain why ...

Web based G-AP training: Practical Use

***4. Did you have any problems using the website?**

- ☐ Yes
- ☐ No

If 'Yes' can you note what the problem(s) were ...

Web based G-AP training: Practical Use

***5. Approximately how much time did you spend on the web based training prior to attending the G-AP training day?**

- ◯ Less than an hour
- ◯ 1-2 hours
- ◯ 2-3 hours
- ◯ 3-4 hours
- ◯ More than 4 hours

***6. How many sessions did you take to complete the training?**

- ◯ I completed the training in one session
- ◯ I completed the training over a few sessions

Web based G-AP training: Content

***7. Was the content of the web based training relevant to your work with patients?**

◯ Yes

◯ No

If 'No' please briefly explain why ...

***8. To what extent do you agree or disagree with the following statements?**

	Disagree	Agree
The case studies helped me to think about using G-AP with patients	◯	◯
The quiz was a good test of my knowledge about G-AP	◯	◯

If you 'Disagree' with any statement, please briefly explain why ...

Web based G-AP training: Content

***9. Was the web based training good preparation for the G-AP training day?**

◯ Yes

◯ No

If 'No' please state main reason why ...

***10. Do you think you will access the web based training at some future point?**

◯ Yes

◯ No

◯ Possibly

If 'No' please state main reason why ...

Wed based G-AP training: Overall view

***11. How would you rate the web based training overall?**

- Very good
- Good
- Fair
- Poor
- Very poor

***12. Would you recommend the web based G-AP training to others?**

- Yes
- No

Why? Please state the main reason ...

The following questions refer to the G-AP training day ...

The G-AP training day: Environment and format

***13. To what extent do you agree or disagree with the following statements?**

	Disagree	Agree
The G-AP training was delivered in a suitable environment		
There were no major distractions that interfered with my learning		
There were enough breaks during the training day		

The G-AP training day: Length

***14. Having one day to complete the training was...**

- Too short
- Too long
- About right

Any comments?

The G-AP training day: Delivery

***15. To what extent to you agree or disagree with the following statements?**

	Disagree	Agree
The G-AP training was well delivered	◯	◯
I was given adequate opportunities to practise what I was learning	◯	◯
I was given adequate opportunities to ask questions	◯	◯
The PowerPoint presentations enhanced my learning	◯	◯
The training handouts were useful	◯	◯

If you 'Disagree' with any statement, please briefly explain why ...

The G-AP training day: Content

***16. Was the content of the G-AP training day relevant to your work with patients?**

◯ Yes

◯ No

If 'No' can you briefly explain why ...

***17. Did the role play sessions help you to think about using the G-AP framework with patients?**

◯ Yes

◯ No

If 'No' please briefly explain why ...

The G-AP training day: Overall views

***18. How would you rate the G-AP training day overall?**

◯ Very good

◯ Good

◯ Fair

◯ Poor

◯ Very poor

***19. Would you recommend this training day to others?**

- ◯ Yes
- ◯ No

Why? Please state the main reason ...

The G-AP training day: Overall views

***20. Was there anything about the training day you thought was particularly helpful?**

- ◯ Yes
- ◯ No

If 'Yes' could you please tell me what ...

***21. Was there anything about the training day that you thought was particularly unhelpful?**

- ◯ Yes
- ◯ No

If 'Yes' could you please tell me what ...

Implementation of G-AP in practice

***22. How confident are you that you will be able to apply what you've learned in practice?**

- ◯ Not at all confident
- ◯ Somewhat confident
- ◯ Very Confident

If 'Not at all confident' please briefly explain why ...

*23. How committed are you to applying what you've learned in practice?

- ◯ Not at all committed
- ◯ Somewhat committed
- ◯ Very committed

If 'Not at all committed' please briefly explain why ...

Final Thoughts

*24. Is there anything else you'd like to say about any aspect of the G-AP training that hasn't been covered?

- ◯ No
- ◯ Yes

If 'Yes' please comment:

About you (optional)

The G-AP training may be more useful to some team members than others. It would be helpful if you could answer the following two questions so that I can check this out.

25. What professional group do you belong to?

- ◯ Physiotherapy
- ◯ Occupational Therapy
- ◯ Speech and Language Therapy
- ◯ Clinical Psychology
- ◯ Nursing
- ◯ Medicine
- ◯ Social Work
- ◯ Dietetics
- ◯ Rehabilitation assistant (any discipline)
- ◯ Day Centre Officer
- ◯ Manager
- ◯ Student

Other (please specify):

26. What is the name of your team/ service?

G-AP Taining Evaluation

Thank you for taking the time to complete this survey - it is very much apprectiated!
