# Supplementary material for: Facilitators and “deal breakers”: a mixed methods study investigating implementation of the Goal setting and action planning (G-AP) framework in community rehabilitation teams
Source: BMC Health Serv Res. 2020 Aug 25;20:791. doi: 10.1186/s12913-020-05651-2 (PMC7447562; doi:10.1186/s12913-020-05651-2)
Supplement: Supplementary file 6 — Additional file 6. [file 12913_2020_5651_MOESM6_ESM.doc]

# Supplementary File 5. Case note review data extraction table

**Stroke Survivor ID: ___________ Key: 1= always 2= sometimes 3= never**

| **Key components of G-AP** | **1** | **2** | **3** | **Comments/ evidence/ examples** |
| --- | --- | --- | --- | --- |
| **Were goals negotiated?** |  |  |  |  |
| **Were person centred goals set?** Were goals written in stroke survivors own words? |  |  |  |  |
| **Were action agreed to meet set goals?** Were action plans written in stroke survivors own words? |  |  |  |  |
| **Were coping plans agreed if barriers identified?** |  |  |  |  |
| **Was confidence to complete action plan measured?** |  |  |  |  |
| **Was performance appraised and feedback given?** |  |  |  |  |
| **Were decisions made about what to do next?** |  |  |  |  |
| **Were subsequent goals set?** |  |  |  |  |
| **Any other relevant information?** |  |  |  |  |
