# Supplementary material for: Facilitators and “deal breakers”: a mixed methods study investigating implementation of the Goal setting and action planning (G-AP) framework in community rehabilitation teams
Source: BMC Health Serv Res. 2020 Aug 25;20:791. doi: 10.1186/s12913-020-05651-2 (PMC7447562; doi:10.1186/s12913-020-05651-2)
Supplement: Supplementary file 7 — Additional file 7. [file 12913_2020_5651_MOESM7_ESM.docx]

**Supplementary File 5. Final Thematic Framework**

| **Themes** | **Sub themes** |
| --- | --- |
| 1. **What helped G-AP implementation** | - Staff liked the concept of G-AP - Staff saw value of G-AP to: ***(i)*** *patients & family,* ***(ii)*** *goal setting practice and* ***(iii)*** *teamwork* - G-AP compatible with other rehabilitation activities - G-AP could be tailored to individual patients - Staff had the right skills - Positive aspects of G-AP training - Positive aspects of G-AP record - Organisational facilitators (Team meetings; Staff/ patient continuity; mentoring opportunities) |
| 1. **What hindered G-AP implementation?** | - Current goal setting practice OK - Potential negative impacts of G-AP on ***(i)*** *patients,* ***(ii)*** *practice* - G-AP not compatible with other rehabilitation activities - Not all staff have the right skills - Issues with G-AP training - Negative aspects of G-AP record - Organisational barriers (Team meetings, Lack of staff/ patient continuity, Not using G-AP with all patient groups; staff turnover) - Difficulty transitioning from training to implementation |
| 1. **Lessons Learned** | - Mapping G-AP to team rehabilitation process - Do not limit G-AP to stroke patients - Tailor G-AP to individual patients - Creating mentoring opportunities - Create G-AP ‘implementation group’ - Improvements to G-AP training - Improvements to G-AP record - Attitudinal change |
