# Supplementary material for: Facilitators and “deal breakers”: a mixed methods study investigating implementation of the Goal setting and action planning (G-AP) framework in community rehabilitation teams
Source: BMC Health Serv Res. 2020 Aug 25;20:791. doi: 10.1186/s12913-020-05651-2 (PMC7447562; doi:10.1186/s12913-020-05651-2)
Supplement: Supplementary file 8 — Additional file 8. [file 12913_2020_5651_MOESM8_ESM.docx]

**Supplementary File 6. G-AP NPT coding framework**

| **Coherence**  **(Sense-making work)**  Meaning & sense making of G-AP by team members | **Cognitive participation**  **(Relational work)**  Team members commitment to & engagement with G-AP | **Collective action**  **(Operational work)**  The work the team and its members do to implement G-AP | **Reflexive monitoring**  **(Appraisal work)**  Ongoing appraisal and tailoring of G-AP within local settings |
| --- | --- | --- | --- |
| **1.1 Differentiation**  Did team members distinguish G-AP from usual practice? | **2.1 Initiation**  Did team members drive G-AP forward? | **3.1 Interactional workability**  Did team members perform the tasks required of G-AP? | **4.1 Systematisation**  Did team members have the information (formal/ informal) to determine how useful G-AP was? |
| **1.2 Communal specification**  Did team members collectively agree about the purpose of G-AP? | **2.2 Enrolment**  Did the team have the capacity & willingness to organise themselves to implement G-AP? | **3.2 Relational integration**  Did team members maintain confidence in their own and other team members work using G-AP? | **4.2 Communal appraisal**  Did team members collectively assess G-AP as worthwhile? |
| **1.3 Individual specification**  Did team members individually understand what the G-AP required of them? | **2.3 Legitimation**  Did team members agree that G-AP should be part of their work? | **3.3 Skill set workability**  Was the ‘work’ of G-AP appropriately allocated within the team? | **4.3 Individual appraisal**  Did team members individually assess G-AP as worthwhile? |
| **1.4 Internalisation**  Did team members recognise G-AP as having added value? | **2.4 Activation**  Did team members continue to support use of G-AP? | **3.4 Contextual integration**  Was G-AP supported by the service & adequately resourced? | **4.4 Reconfiguration**  Did team members modify their work in response to their appraisal of G-AP? |
