# Supplementary material for: Facilitators and “deal breakers”: a mixed methods study investigating implementation of the Goal setting and action planning (G-AP) framework in community rehabilitation teams
Source: BMC Health Serv Res. 2020 Aug 25;20:791. doi: 10.1186/s12913-020-05651-2 (PMC7447562; doi:10.1186/s12913-020-05651-2)
Supplement: Supplementary file 9 — Additional file 9. [file 12913_2020_5651_MOESM9_ESM.docx]

**Supplementary File 7. Mapping of themes to NPT constructs**

|  | **Coherence** | **Cognitive**  **Participation** | **Collective**  **Action** | **Reflexive**  **Monitoring** |
| --- | --- | --- | --- | --- |
| **Facilitators** | - Staff liked concept of G-AP; saw value in it - Training helped staff make sense of G-AP | - Staff had the right skills and attitudes to implement G-AP | - **Organisational Facilitators** - G-AP compatible with other rehabilitation activities - Continuity between staff and patients - Opportunity to practice and consolidate skills - G-AP can be tailored to individual stroke survivors | - Perceived positive G-AP impact on patients, family and practice e - Having a mentor - Positive aspects of G-AP record - Reconfiguration of team meetings |
| **Barriers** |  | - G-AP ‘champion’ left - Staff turnover - Lack of leadership support | - **Organisational barriers** - Scheduling of sessions; lack of continuity - Current team processes don’t ‘fit’ with G-AP - Delay between G-AP training and implementation - Not using G-AP with all patients - **G-AP Training Issues** - More focus on practical use of G-AP record - Staff not supported to consider local implementation | - **Issues with G-AP record** - Record not accessible to stroke survivors with aphasia - **Potential negative impact on practice and patients** - Focus on behavioural rather than emotional goals - Some patients too emotionally overwhelmed - Current practice satisfactory |
| **Lessons Learned** |  | - Need for attitudinal change |  | - Improvements to G-AP training and G-AP record - Plan, monitor and tailor local G-AP implementation - Use G-AP with all patients (in teams seeing a mixed patient group) |
